# MADS-Box Genes Are Key Components of Genetic Regulatory Networks Involved in Abiotic Stress and Plastic Developmental Responses in Plants

**DOI:** 10.3389/fpls.2019.00853

**Published:** 2019-07-10

**Authors:** Natalia Castelán-Muñoz, Joel Herrera, Wendy Cajero-Sánchez, Maite Arrizubieta, Carlos Trejo, Berenice García-Ponce, María de la Paz Sánchez, Elena R. Álvarez-Buylla, Adriana Garay-Arroyo

**Affiliations:** ^1^Laboratorio de Genética Molecular, Epigenética y Desarrollo de Plantas, Instituto de Ecología, Universidad Nacional Autónoma de México, Mexico City, Mexico; ^2^Postgrado en Recursos Genéticos y Productividad-Fisiología Vegetal, Colegio de Postgraduados, Texcoco, Mexico; ^3^Centro de Ciencias de la Complejidad, Universidad Nacional Autónoma de México, Mexico City, Mexico; ^4^Postgrado en Botánica, Colegio de Postgraduados, Texcoco, Mexico

**Keywords:** MADS-box genes, abiotic stress, development, growth, flowering

## Abstract

Plants, as sessile organisms, adapt to different stressful conditions, such as drought, salinity, extreme temperatures, and nutrient deficiency, via plastic developmental and growth responses. Depending on the intensity and the developmental phase in which it is imposed, a stress condition may lead to a broad range of responses at the morphological, physiological, biochemical, and molecular levels. Transcription factors are key components of regulatory networks that integrate environmental cues and concert responses at the cellular level, including those that imply a stressful condition. Despite the fact that several studies have started to identify various members of the MADS-box gene family as important molecular components involved in different types of stress responses, we still lack an integrated view of their role in these processes. In this review, we analyze the function and regulation of MADS-box gene family members in response to drought, salt, cold, heat, and oxidative stress conditions in different developmental processes of several plants. In addition, we suggest that MADS-box genes are key components of gene regulatory networks involved in plant responses to stress and plant developmental plasticity in response to seasonal changes in environmental conditions.

## 1. Introduction

Plants face seasonal fluctuations and stressful environmental conditions, involving alterations in light quality and regimes (e.g., short or long days), precipitation, nutrient availability, temperature, drought, flooding, salinity, and UV exposure, among others. Stressful conditions affect multiple metabolic pathways that, in turn, can induce and integrate different intrinsic responses affecting developmental and plant morphogenetic responses. These responses allow plants to survive and adapt to a plethora of environments.

Plant development and their responses to natural changing environments and stressful conditions are often regulated by complex regulatory networks that include various types of molecular components, such as transcription factors (TFs), regulatory RNAs, and enzymes (Fujita et al., 2011; Nakashima and Yamaguchi-Shinozaki, 2013; Janiak et al., 2016; Wils and Kaufmann, 2017; Cho, 2018; Gruszka, 2018; Sarvepalli et al., 2019). In the context of regulatory networks, TFs are particularly relevant because they regulate the expression of multiple target genes and their loss or gain of function may lead to dramatic phenotypic alterations or modified plastic responses to environmental changes (Goto and Meyerowitz, 1994; Jack et al., 1994; Kramer et al., 1998; Honma and Goto, 2001; Ditta et al., 2004; Sakuma et al., 2006; Hernández-Hernández et al., 2007; Nelson et al., 2007; Welch et al., 2007).

MADS-domain TFs are key members of regulatory networks underlying multiple developmental pathways in plants, animals, and fungi (Goto and Meyerowitz, 1994; Jack et al., 1994; Honma and Goto, 2001; Pelaz et al., 2001; Messenguy and Dubois, 2003; Nadal et al., 2003; Tapia-Lopez et al., 2008; Garay-Arroyo et al., 2013; Cao et al., 2016; Thangavel and Nayar, 2018). The MADS acronym was formed by the initials of the first four MADS-domain proteins discovered: M for MINICHROMOSOME MAINTENANCE FACTOR 1 from *Saccharomyces cerevisiae*, A for AGAMOUS (AG) from *Arabidopsis thaliana* (from now on, Arabidopsis), D for DEFICIENS from *Antirrhinum majus*, and S for Serum Response Factor (SRF) from *Homo sapiens* (Norman et al., 1988; Passmore et al., 1988; Jarvis et al., 1989; Schwarz-Sommer et al., 1990; Sommer et al., 1990; Yanofsky et al., 1990). The function of MADS-domain proteins has been widely studied in different organisms; these proteins participate in different developmental processes in plants (Smaczniak et al., 2012a), in neural signal transmission, muscle development, and tumor occurrence in humans (Cao et al., 2016), and in osmotic stress response and cell survival in the stationary phase in yeast (Nadal et al., 2003).

A gene duplication gave rise to two MADS-domain protein lineages before the divergence of plants and animals; these lineages are easy to identify due to a strong conservation of the MADS and other protein domains: The Type I or *SRF*-like genes and Type II or *MEF2*-like genes (*MYOCYTE ENHANCER FACTOR 2*) (Alvarez-Buylla et al., 2000; Becker and Theißen, 2003). The Type II lineage, also called MIKC-type in plants, has been subdivided into MICK^c^ and MICK^*^ groups. Furthermore, MIKC^c^ contains 13 different gene subfamilies or clades based on phylogenetic studies (Theißen et al., 1996; Becker and Theißen, 2003; Parenicová et al., 2003). During land plant evolution, the number and functional diversity of MADS-box genes increased due to multiple gene and genome duplications, reaching 108 members (see Table S3 from Parenicová et al., 2003) in Arabidopsis (Becker and Theißen, 2003; Parenicová et al., 2003; Kaufmann et al., 2005; Gramzow et al., 2010; Fan et al., 2013). Thus, MADS-box genes are widely distributed in a taxonomically broad range of monocot and dicot plant species (Table S1). It has been proposed that changes in MADS-box gene structure, expression, and function have been a major cause for innovations in development during land plant evolution (Theißen et al., 1996; Zahn et al., 2006).

MADS-domain proteins are able to bind DNA as homo or heterodimers together with other MADS-domain proteins or with other proteins as part of different protein complexes (Schwarz-Sommer et al., 1992; Goto and Meyerowitz, 1994; Davies et al., 1996; Huang et al., 1996; Mizukami et al., 1996; Riechmann et al., 1996; Egea-Cortines et al., 1999; Pelaz et al., 2000; Honma and Goto, 2001; Sridhar et al., 2006; Tröbner et al., 2018) and function as tetramers in order to regulate transcription of their target genes (Pelaz et al., 2000; Honma and Goto, 2001; Theißen and Saedler, 2001; Sridhar et al., 2006; Brambilla et al., 2007; Immink et al., 2009; Melzer and Theißen, 2009; Smaczniak et al., 2012b). MADS-domain protein interactions, either with members of the same family or with other proteins, could explain their specificity and their ability to orchestrate different developmental programs that respond to external and internal signals such as hormones (Sridhar et al., 2006; Brambilla et al., 2007; Verelst et al., 2007; Hill et al., 2008; Liu et al., 2009; Kaufmann et al., 2010; Smaczniak et al., 2012b; Han et al., 2016). Besides, MADS-domain proteins have thousands of target genes, as shown in different studies (Kaufmann et al., 2009, 2010; Zheng et al., 2009; Deng et al., 2011; Schlesinger et al., 2011; Sullivan et al., 2011). Finally, it is of great interest to understand the MADS-domain proteins' interactome along the diverse developmental pathways they are involved in, and how these interactions could modify gene regulation and, thus, morphogenesis (Sablowski, 2010).

In Arabidopsis, MADS-box genes participate in diverse developmental processes such as meristem specification, flowering transition, seed, root and flower development, and fruit ripening (Smaczniak et al., 2012a). Their function in flower development has been deeply studied and summarized in many excellent reviews (Smaczniak et al., 2012a; Yan et al., 2016; Bartlett, 2017; Bloomer and Dean, 2017; Whittaker and Dean, 2017; Callens et al., 2018; Theißen et al., 2018). Moreover, several MADS-box genes have been implied in plant responses to different abiotic stress conditions using both genomic and functional genetic approaches (Table S1), but we are lacking an integrative view of these studies.

**Figure 1:**
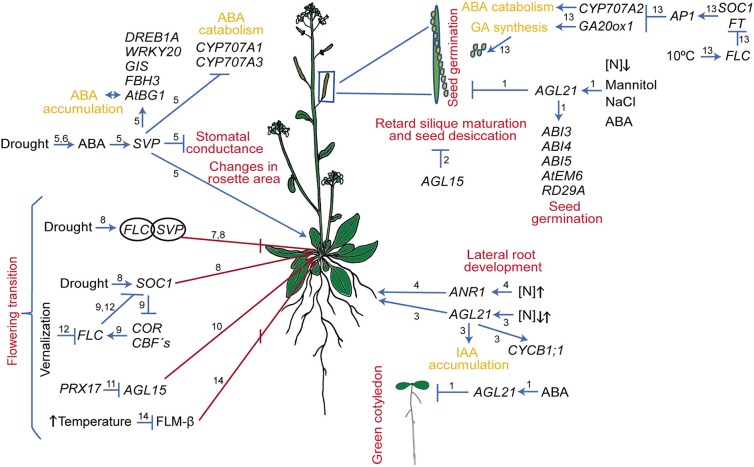
Regulatory circuits mediated by MADS-box genes in response to abiotic stress in *Arabidopsis thaliana*. The different developmental processes affected are indicated in red. Arrows and bar lines indicate induction and repression, respectively. Numbers on the arrows and/or bars indicate the reference where the data were obtained: (1) Yu et al. (2017); (2) Fang and Fernandez (2002); (3) Yu L.-H. et al. (2014); (4) Zhang and Forde (1998); Gan et al. (2012); (5) Bechtold et al. (2016); (6) Wang et al. (2018); (7) Lee et al. (2007); Li et al. (2008); (8) Riboni et al. (2013, 2016); (9) Seo et al. (2009); (10) Fernandez et al. (2000); (11) Zheng et al. (2009); Cosio et al. (2017); (12) Bloomer and Dean (2017); (13) Chiang et al. (2009); (14) Sureshkumar et al. (2016) and Lutz et al. (2017).

**Figure 2:**
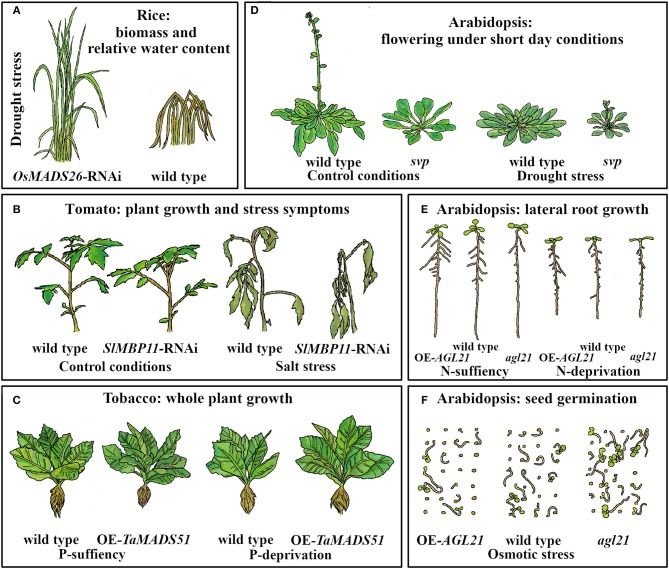
MADS-box genes are involved in abiotic stress response in different developmental processes in several plants. **(A)** *OsMADS26* is a negative regulator of drought stress tolerance in rice. The cartoon represents downregulation of *OsMADS26* (*OsMADS26*-RNAi) and wild type plants in conditions of water stress for 18 days followed by 15 days of re-watering (Khong et al., 2015). **(B)** *SlMBP11* is required in order to give more tolerance for salt stress to tomato plants. Plants are more affected by salt stress condition (100 mM NaCl) when this gene is downregulated (*SIMBP11*-RNAi) (Guo et al., 2016). **(C)** Overexpression (OE) of *TaMADS51* in transgenic tobacco plants improves plant growth under phosphorous (P)-deprivation (Shi et al., 2016). In Arabidopsis: **(D)** *SVP* repress the onset of the flowering in drought escape response at short day conditions. Cartoons are representative of wild type plants (16 weeks old) and *svp* mutants (8 weeks old) subjected to control conditions or drought regime (Riboni et al., 2013). **(E)** *AGL21* is important for LR development in control conditions and under nitrogen (N)-deprivation (Yu L.-H. et al., 2014). **(F)** *AGL21* function as a negative regulator of seed germination under osmotic stress conditions (300 mM mannitol) (Yu et al., 2017).

**Figure 3:**
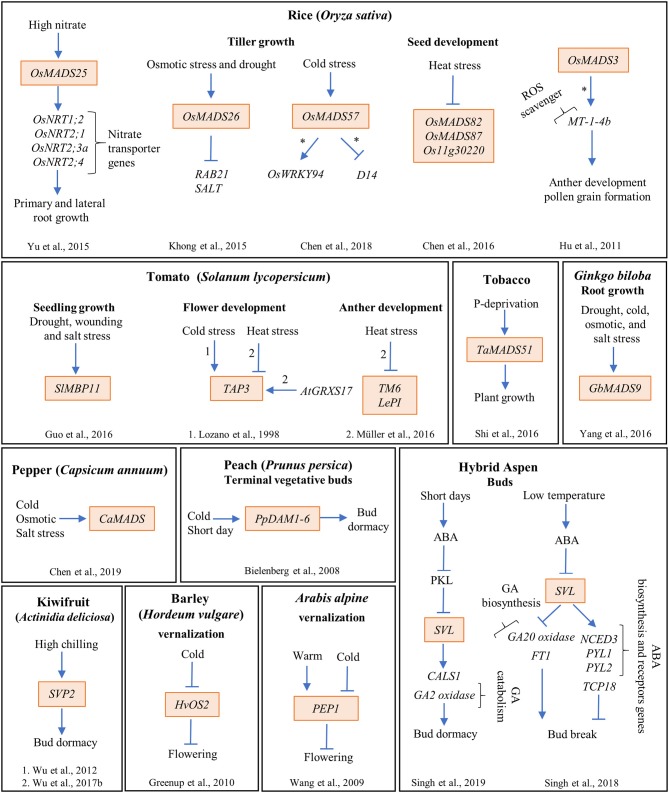
The MADS-box genes participate in diverse genetic interactions that integrate the environmental signals to several development processes in different plant species. The MADS-box genes are highlighted in orange boxes; arrows and bar lines indicate induction and repression, respectively; asterisk (*) means direct regulation; and the numbers on the arrows indicate the reference where the data were obtained.

The aim of this review is to integrate and analyze the available information regarding the participation of MADS-domain proteins in regulatory networks involved in abiotic stress and developmental plastic responses, primarily in Arabidopsis (see Figures 1, 2), but also in other plant species (see Figures 2A–C, 3).

## 2. MADS-Domain Transcription Factors' Role in Developmental Plasticity Responses to Stressful Conditions

Plant development is highly plastic, allowing plants to survive and adapt to different environments (Ludlow, 1980; Zhu, 2016). Stressful conditions may trigger multiple endogenous signaling pathways that, in turn, can induce an adjustment of metabolic and morphogenetic processes. Among the abiotic stress factors that greatly affect plant development in a stage-specific manner, from seed germination to reproduction, are drought, high salinity, low or high temperatures, and nutritional deficiencies (Levitt, 1985; Manavalan et al., 2009; Jogaiah et al., 2013; Osakabe et al., 2014; Zhu, 2016). During abiotic stress conditions such as drought or heat, the reactive oxygen species (ROS) concentration can be augmented dramatically and surpass the cell's antioxidant system, thus generating oxidative stress (Choudhury et al., 2017). To cope with oxidative stress, cells have developed multiple antioxidant molecules that can be classified as enzymatic or non-enzymatic according to their nature (Birben et al., 2012). The former includes many enzymes that catalyze the reduction of ROS such as catalase, peroxidase, superoxide dismutase, peroxiredoxin, and glutaredoxins that use glutathione as a cofactor (Mittler et al., 2004). Non-enzymatic ROS detoxification molecules include multiple reducing agents like flavonoids, carotenoids, and glutathione; these compounds reduce ROS and prevent them from oxidizing other cellular components (Maurino and Flügge, 2008). In this section, we will review how MADS-domain TFs participate in abiotic stress responses during vegetative growth, flowering, and root and seed development.

### 2.1. Development of Vegetative Organs Under Stress Conditions

Drought is one of the major factors that negatively affects plant growth and survival and plants have developed different adaptations to withstand water limitation (Boyer, 1982; Davies and Zhang, 1991; Comstock, 2002; Chaves and Oliveira, 2004). Water stress can be induced not only by drought but also by cold and high salinity conditions since they reduce the water potential, generating low water availability. Additionally, the phytohormone abscisic acid (ABA) is critical to withstanding stress and integrating stress signals. Therefore, plants are constantly regulating their ABA content in response to different external and internal conditions (Vishwakarma et al., 2017; Jurkiewicz and Batoko, 2018). Drought, cold, and high salinity induce common response mechanisms that include ABA-dependent and ABA-independent signaling transduction pathways (Bartels and Sunkar, 2005; Yoshida et al., 2014a).

Rice (*Oryza sativa*) is one of the most important crops in the world and its plantations are especially susceptible to drought conditions. It has been reported that several MADS-box genes are differentially expressed during drought stress in rice (Table S1). For example, the expression of *OsMADS26*, the rice *AGL12* ortholog, is enhanced by mannitol, a compound that induces osmotic stress by mimicking drought stress conditions (Lee et al., 2008). OsMADS26 acts as a regulator of stress-related responses such as drought or pathogen infections (Khong et al., 2015). Interestingly, overexpression (OE) of this gene, either in rice or in Arabidopsis plants grown under control conditions, causes a severe stress phenotype that kills most plants. The majority of the survivors, show reduced root/shoot growth, sterility, root curling, and a pale green coloration (Lee et al., 2008). Accordingly, *OsMADS26-GR* lines induced with different concentrations of dexamethasone show curly leaves, short shoots and roots, and roots with a purple pigmentation. The phenotypes observed in the OE lines (either constitutive or inducible) correlated with those observed in plants exposed to stressful conditions (Lee et al., 2008). In contrast, Khong et al. (2015) did not observe any severe phenotypic alteration in *OsMADS26* OE plants grown under control conditions.

Moreover, Lee et al. (2008) reported that *OsMADS26* loss of function and wild type plants exhibited similar behaviors under stress conditions. However, Khong et al. (2015) found that, under water deficit, loss of function lines of *OsMADS26* (*OsMADS26*-RNAi) showed higher biomass and yield, increased plant capacity to maintain both chlorophyll a and b content and leaf relative water content (RWC), and improved recovery potential after re-watering, compared to wild type plants (Figure 2A). In addition, Lee et al. (2008) reported that OsMADS26 positively regulates many genes involved in stress-related processes, especially genes that participate in ROS homeostasis such as NADPH-oxidase, peroxidase, and oxidoreductases. Nevertheless, these results have little overlap with those from Khong et al. (2015), who found that OsMADS26 negatively regulates drought, salt, and ROS responsive genes such as *RESPONSIVE TO ABA21*, encoding a rice dehydrin, *SALT STRESS-INDUCED PROTEIN* and the ROS scavenging enzyme Peroxidase 22 (Figure 3).

Discrepancies between these two groups' results may be explained either by disparities in the expression levels of *OsMASDS26*: no expression in the knockout mutant (Lee et al., 2008) vs low expression in the *OsMADS26*-RNAi line (Khong et al., 2015), or by the different genetic backgrounds employed in the studies. Despite this, it seems clear that OsMADS26 plays a fundamental role as a stress-signal integrator and that systemic approaches are still needed to unravel the participation of OsMADS26 in the regulatory network underlying water stress responses.

Other studies made in rice determined that OsMADS57, one of the five MADS-domain proteins belonging to the AGL17 clade, functions as a promoter of tolerance to cold stress (Arora et al., 2007). The gene is induced by salt, drought, abscisic acid, and chilling. In addition, it was shown that the OE line had a higher survival rate when exposed to chilling temperatures (4°C) than wild type plants, and the opposite was true for the loss of function line. Moreover, OsMADS57 directly represses the expression of *Dwarf14*, a gene involved in axillary bud development, while it directly activates the expression of *OsWRKY94*, which participates in several stress responses and various plant developmental processes. Interestingly, both binding and activation of *OsWRKY94* by OsMADS57 were shown to be temperature-dependent, suggesting that OsMADS57 may function as an on-off switch to change from transcriptional repression at normal temperatures to activation at chilling temperatures (Figure 3; Chen et al., 2018).

In Arabidopsis, the MADS-domain TF SHORT VEGETATIVE PHASE (SVP) provokes modifications in some developmental processes and gas exchange function in response to progressive drought stress (Figure 1; Bechtold et al., 2016). In addition, despite a significant reduction in stomatal conductance under well-watered conditions and drought stress, *svp* loss of function mutants exhibit elevated water loss and maintain substantial photosynthetic CO_2_ assimilation rate throughout the drying period due to persistent rosette growth in comparison to wild type plants (Bechtold et al., 2016; see Figure 2D). Furthermore, it was shown that SVP regulates the expression of eight TFs that respond to drought; two of these, *DEHYDRATION RESPONSE ELEMENT B1A* and *FLOWERING BHLH 3*, are particularly interesting because they are involved in early responses to drought and osmotic stress, such as stomatal opening regulation (Kasuga et al., 2004; Yoshida et al., 2014b; Bechtold et al., 2016).

In addition, it was also reported that *SVP* is induced by drought stress and functions as a positive regulator of drought resistance via ABA homeostasis. Wang et al. (2018) showed that *SVP* loss of function mutants are sensitive to drought stress conditions, while the OE lines are tolerant. Moreover, SVP affects genes involved in ABA function as it directly downregulates the expression of *CYP707A1* and *CYP707A3*, the ABA 8-hydroxylase genes that participate in ABA catabolism in Arabidopsis leaves. SVP also upregulates *AtBG1*, a β-glucosidase gene involved in the hydrolysis of ABA-glucose ester (ABA-GE) into ABA, an important step for the accumulation of active ABA. All these data suggest that SVP is an important component of a regulatory network involved in drought responses (Figure 1; Riboni et al., 2013; Bechtold et al., 2016).

In tomato (*Solanum lycopersicum*), the expression of *SlMBP11* (an *AGL15* ortholog) is induced by salt stress, dehydration, and wounding (Figures 2B, 3). Knockdown lines of this gene (*SlMBP11*-RNAi) are more sensitive to salt stress conditions (100 mM NaCl) than wild type plants (Figure 2B), showing reduced fresh weight as well as reduced root growth in both post-germination and 5-week-old seedlings. Moreover, these plants also showed lower RWC and chlorophyll content, alterations associated with sensitivity to salt stress (Guo et al., 2016). Additionally, several parameters related to oxidative damage such as relative electrolyte leakage and malondialdehyde content are higher in the *SlMBP11*-RNAi plants, suggesting that this line is having an oxidative damage. Contrary to what happened with the loss of function mutant of *SlMBP11* in 5-week-old seedlings, the OE of this MADS-box gene confers tolerance to salt stress, has a higher RWC, accumulates greater amounts of chlorophyll content and less amounts of malondialdehyde, and shows diminished relative electrolyte leakage (Guo et al., 2016).

Interestingly, *SIMBP8*, a gene closely related to *SIMBP11*, has the opposite effect on salt tolerance in tomato. This MADS-box gene is a negative regulator of drought and high salinity stress responses in this plant as *SlMBP8*-RNAi seedling root and shoot growth are less inhibited than those of wild type seedlings growing under salt stress conditions (100 mM NaCl) and drought stress in a 5-week-old seedlings (Yin et al., 2017). These authors also found that the RNAi line, as the OE line of *SlMBP11*, has higher levels of RWC and chlorophyll content, and less amounts of relative electrolyte leakage and malondialdehyde, both in salt and drought stress (Guo et al., 2016; Yin et al., 2017).

In pepper (*Capsicum annuum*), it was shown that *CaMADS* (a MADS-box gene from the AGL2/SEP1 clade) is induced by various stress conditions (Figure 3; Table S1). Downregulation of this gene in pepper originates plants more sensitive to cold, salt stress, and mannitol treatment, that show increased malondialdehyde levels and electrolyte leakage, as well as lower levels of chlorophyll than wild type plants. Moreover, the OE of *CaMADS* in Arabidopsis plants confers a higher tolerance to cold, salt, and osmotic stress, and contributes to a better recovering capacity of the plants after cold stress conditions in comparison to wild type plants (Chen et al., 2019).

Finally, the availability of mineral nutrients is another of the most limiting resources of plant growth. Phosphorous (P) is an essential nutrient for plant growth that can alter the root system architecture (Gruber et al., 2013; Rellán-Álvarez et al., 2016; Shahzad and Amtmann, 2017). In Arabidopsis, P deficiency inhibits primary root (PR) elongation while it increases lateral root (LR) density and length (Svistoonoff et al., 2007). Furthermore, in wheat (*Triticum aestivum*), nine out of 54 MADS-box genes are differentially regulated under P-deprivation conditions (Shi et al., 2016). One of them, *TaMADS51* that is induced under P deprivation (12 μmol/L P), was used for functional analysis in tobacco plants grown under hydroponic conditions (Figures 2C, 3). The study showed that the OE of this MADS-box gene improves plant growth and increments plant biomass and P accumulation as well as antioxidant enzymatic activities only under P deprivation growth conditions (Figures 2C, 3; Shi et al., 2016).

### 2.2. Root Development

Root system architecture, i.e., the spatial arrangement of an entire root system, changes plastically in response to various environmental cues to ensure water and nutrient uptake to sustain growth and survival (Shahzad and Amtmann, 2017). In Arabidopsis, it is known that at least 50 MADS-box genes are expressed in the root (Rounsley et al., 1995), but their participation in root development in response to abiotic stress is largely unknown.

*GbMADS9* is a *Ginkgo biloba* MADS-box gene involved in post-germination root growth in response to abiotic stress tolerance (Yang et al., 2016); it is an ortholog of the B sister-class genes of Arabidopsis. *GbMADS9* is upregulated in response to salt, drought and cold stresses (Figure 3); and Arabidopsis OE lines of this gene exhibited longer roots than those of wild type plants after 15 days under high osmotic stress (400 mM of mannitol). Additionally, malondialdehyde was lower, while the chlorophyll and proline levels were higher in these plants compared to those from wild type plants. Proline is an amino acid that may enhance stress tolerance because it works as an osmolyte, a metal chelator, an antioxidative defense molecule, and a signaling molecule (Hayat et al., 2012). Finally, enhanced tolerance to osmotic conditions was associated with improved superoxide dismutase and catalase antioxidant enzymatic activities in these OE lines (Yang et al., 2016). These data suggest that the increase tolerance of the OE lines of *GbMADS9*, could be dependent on the proline content and the enhance of antioxidant activities.

Nitrogen (N) is an inorganic nutrient essential for plant growth and development; it alters the root system architecture depending on the N source as well as on its concentration (Gruber et al., 2013). Vidal et al. (2010) found that Arabidopsis PR growth is inhibited while LR density is increased under 5 mM KNO_3_. In addition, Zhang et al. (1999) found a gradual increase of LR length in roots locally exposed to increasing concentrations of KNO_3_. It has also been reported that when the plants are permanently exposed to high NO_3_- concentrations (≥10 mM), LR production and initiation is inhibited (Zhang and Forde, 1998). These studies suggested that direct root contact to a NO_3_- source stimulates LR meristem activity, while a high amount of NO_3_- absorbed by the plant could have an inhibitory effect on it (Zhang and Forde, 1998; Zhang et al., 1999). Next, we will review in detail the involvement of MADS-domain proteins in root system architecture changes due to alterations in nitrogen supply.

*ARABIDOPSIS NITRATE REGULATED 1* (*ANR1/AGL44*) is a well-known positive regulator of LR development in response to nitrate availability (Zhang and Forde, 1998). *ANR1* antisense and co-suppressed lines show inhibition of LR elongation when exposed to high N concentrations (Zhang and Forde, 1998). Interestingly, LR growth in these lines is no longer responsive to the stimulatory effect of NO_3_- supplied locally, suggesting that *ANR1* participates in the NO_3_- signal transduction pathway that modulates LR growth (Zhang and Forde, 1998). Besides, the OE of *ANR1* in plants results in a higher LR density as well as longer LRs either under control or high N conditions (Gan et al., 2012). Accordingly, the OE in Arabidopsis of one of the five rice *ANR1* orthologs, *OsMADS25*, induced LR formation, PR and LR length increments, and gains in root and shoot fresh weight in the absence of NO_3_-, compared to wild type plants. This effect is enhanced in response to increasing NO_3_- levels (Figure 3; Yu et al., 2015). Additionally, two rice OE lines of *OsMADS25* showed longer PR and LRs and a higher density of LRs than wild type plants. As expected, this gene's RNAi lines showed shorter PR and LRs and smaller LR numbers in comparison to wild type plants under high nitrate (Yu et al., 2015). Moreover, a previous study of the one published in 2015 by Yu et al., showed that diverse N sources have contrasting effects over *OsMADS25* expression levels: the gene is upregulated by KNO_3_ and NH_4_NO_3_, whereas it is downregulated by NH_4_Cl (Table S1; Yu C et al., 2014).

*AGAMOUS LIKE21* (*AGL21*) is a MADS-box gene highly expressed in the vascular tissue of the Arabidopsis PR and in LR primordia (Yu L.-H. et al., 2014). This gene is induced by different environmental stresses and plant hormone treatments (Figure 1 and Table S1), suggesting participation in root plasticity (Yu L.-H. et al., 2014). *AGL21* OE lines produce higher numbers and longer LRs both under N-deficient and N-rich conditions (20 mM KNO_3_ and 20 mM NH_4_NO_3_), while the loss of function mutant produces fewer and shorter LRs than wild type plants in either N-growing conditions. This data revealed that LR responses to nitrogen availability in *AGL21* mutant lines, are not directly proportional to the concentration used. Finally, AGL21 positively regulates cell division in LR primordia and LRs, but PR length was not affected either in *AGL21* OE or in loss of function lines (Figure 2E; Yu L.-H. et al., 2014).

### 2.3. Involvement of MADS-Box Genes in Flower Development

Tomato flowers are very sensitive to low temperatures, displaying three different phenotypes, or combinations of them, after facing cold stress: floral organ homeotic changes, modifications in organ number, and differences in the pattern of organ fusion (Lozano et al., 1998). Interestingly, several MADS-box genes, including *TOMATO APETALA3*, are highly induced under cold stress conditions (Table S1), suggesting that differential expression of MADS-box genes could be responsible for these plastic phenotypical alterations (Lozano et al., 1998).

Stamens are the male reproductive organs, consisting of a filament and an anther in which microspores are produced. It has been shown that temperature influences sexual reproduction (Kim et al., 2001; Bokszczanin, 2013), specifically, the development and functioning of male gametophyte in monocot and dicot species (Müller et al., 2016). Stamen specification is regulated by a tetramer integrated by the MADS-domain proteins AP3, PI, AG, and SEP (Theißen and Saedler, 2001).

Tomato develops many different stamen phenotypes under continuous mild heat conditions (32°C day, 26°C night). They include loss of pollen viability and deformation of some of the anthers into pistil-like structures. The frequency of appearance of these phenotypes is not constant, it increases as temperature rises. The homeotic transformation of the anthers suggested that the expression patterns of B and C class genes from tomato should be analyzed: two *AP3* genes, *TAP3* and *TOMATO MADS BOX GENE6* (*TM6*), two *PI* genes, *LePISTILLATA* (*LePI*) and *TOMATO PISTILLATA*, and two C-class genes *TOMATO AGAMOUS1* and *TAG-LIKE1*. Müller et al. (2016) showed that, under mild heat conditions, the expression of *TM6, TAP3*, and *LePI* is repressed in anthers while the other MADS-box genes do not change their expression (Figure 3). They decided to use a *TM6*-RNAi allele and observed weak anther-to-pistil conversions under control conditions (25°C day, 19°C night) while, under a regimen of mild heat conditions (32°C/26°C day/night) several phenotypes appeared, such as strong anther deformation, reduced male fertility, and less pollen production with lower viability than wild type plants, resembling plants growing at high temperatures. These data demonstrate that reduction in the expression of the MADS-box gene *TM6* mimics phenotypes obtained with high-temperature growth conditions in tomato plants. Interestingly, plants overexpressing *AtGRXS17*, a glutaredoxin that confers heat tolerance when overexpressed, show fewer anther deformities under mild heat conditions than wild type plants. Moreover, these plants also show an increment in the expression of *TAP3* (Müller et al., 2016).

In rice, *OsMADS3*, an *AG* ortholog, is expressed in the tapetum and in microspores during late anther development stage. A knockout line of this gene *(osmads3-3)* shows homeotic transformation of stamens into lodicules (Yamaguchi et al., 2006). In another study, null expression mutant, *osmads3-4*, is male sterile and exhibits defects in pollen development due to oxidative stress, suggesting that OsMADS3 regulates anther development (Hu et al., 2011). Using chromatin immunoprecipitation and electrophoretic mobility shift assay, it (Hu et al., 2011) showed that OsMADS3 regulates directly the expression of *MT-1-4b*, a gene encoding a metal binding protein that functions as a ROS-scavenger. Furthermore, silencing of *MT-1-4b* by an artificial miRNA (*MT-1-4b*-amiRNA) provoked defects in anther's development and reduced mature pollen grain formation (Figure 3). Finally, these authors also showed that OsMADS3 affects the expression of many other ROS-scavenging enzymes, suggesting that this MADS-box gene regulates male reproductive development, in part, through ROS homeostasis. It has been demonstrated that ROS participate in multiple developmental processes like root and flower development, meristem specification, and seed germination (Tsukagoshi et al., 2010; Schippers et al., 2016; Zeng et al., 2017). Under normal conditions, ROS production in plants is low, while under stress conditions, it is highly induced and may provoke cellular damage. However, it has also been suggested that ROS molecules may play a role in stress signaling (Price and Hendry, 1991; Mittler, 2002).

### 2.4. Seed Development

Seeds enclose embryonic plants and are essential to determining when and where plants should establish themselves. Seed germination starts with initial water uptake by a quiescent dry seed and terminates with the elongation of the embryonic axes and the emergence of the embryonic root (Bassel et al., 2011). This developmental process is highly regulated by temperature and water availability. Also, under adverse conditions, seed germination is arrested (dormancy), allowing the plant to survive. Two plant hormones are important in this developmental process as ABA delays germination, whereas gibberellic acid (GA) promotes it (Shu et al., 2016).

In Arabidopsis, Yu et al. (2017) showed that *AGL21*, besides its role in LR development, functions as a negative regulator of seed germination under osmotic stress conditions. While OE of *AGL21* affects germination rate and seeds become hypersensitive to salt stress (150 mM NaCl), to severe osmotic stress (300 mM mannitol), and to ABA, the germination rate of the *AGL21* loss of function lines is less affected than that of wild type plants under these stressful conditions (Figures 1, 2F). Interestingly, the authors demonstrated that *AGL21* is involved in ABA signaling as it directly regulates the expression of *ABA INSENSITIVE 5* (*ABI5*); furthermore, they also showed that *AGL21*-regulated seed germination depends on *ABI5*.

Another gene involved in seed development in Arabidopsis is *AGL15*. The OE lines of this gene retard silique maturation and seed desiccation. Besides, seeds have a higher water potential for a longer period of time than seeds from control plants (Figure 1; Fang and Fernandez, 2002).

In rice, it has been shown that several Type I MADS-box genes participate in early seed formation during the syncytial stage of development that, among other things, determines seed size (Folsom et al., 2014). Chen et al. (2016) showed that the expression of three rice MADS-box genes, *OsMADS82, OsMADS87*, and *Os11g30220*, is reduced upon heat treatment (from mild 35°C/30°C to severe 39°C/34°C day/night) for 48 h after fertilization (Figure 3). Also, rice seed size and seed viability are reduced during heat treatment because the transition from syncytium to cellularization accelerates. Thus, the authors decided to study the function of one of these genes, *OsMADS87*, in rice mutant lines (Chen et al., 2016). They found that decreasing *OsMADS87* expression through RNAi transgenic rice lines growing under normal conditions accelerates endosperm cellularization and reduces seed size. On the other hand, the OE of this gene does not change endosperm cellularization but produces larger seeds at maturity compared to wild type seeds. In heat stressed plants (35°C) the OE and the wild type lines show reduced seed size compared to control conditions, while the RNAi lines are not further affected (Chen et al., 2016). These data suggest that OsMADS87 participates in endosperm cellularization and seed size in response to heat stress.

### 2.5. Flowering Time

Flowering is a developmental stage that involves the transition from vegetative growth to a reproductive phase where flowers are produced; this developmental stage is tightly regulated by both endogenous and environmental cues (Hepworth and Dean, 2015; Kazan and Lyons, 2016). Complex regulatory networks underlie this transition, where MADS-box genes play central roles not only in the transition itself, but also in the shoot apical meristem homeostasis and floral organ identity (Srikanth and Schmid, 2011; Andrés and Coupland, 2012; Bloomer and Dean, 2017; Whittaker and Dean, 2017; Wils and Kaufmann, 2017).

Flowering is regulated by several environmental factors including photoperiod, light quality, and temperature. It is also regulated by different types of stress conditions such as drought, temperature, oxidative, and salt stress (Blázquez and Weigel, 2000; Onouchi et al., 2000; Samach et al., 2000; Blázquez et al., 2003; Moon et al., 2003; Balasubramanian et al., 2006). In this section, we will review how MADS-box genes participate in the coordination of the response to stress conditions during the vegetative to flowering transition.

In Arabidopsis, flowering time is determined by the expression of the so-called floral pathway integrators (FPIs), including *LEAFY, SUPPRESSOR OF OVEREXPRESSION OF CONSTANS 1* (*SOC1*), and *FLOWERING LOCUS T* (*FT*) (Blázquez and Weigel, 2000; Lee et al., 2000; Onouchi et al., 2000; Samach et al., 2000; Moon et al., 2003, 2005). These genes are antagonistically regulated by two TFs: *CONSTANS* (*CO*), encoding a zinc finger protein, and *FLOWERING LOCUS C* (*FLC*), encoding a MADS-box TF (Michaels and Amasino, 1999; Lee et al., 2000; Samach et al., 2000). During the transition to flowering, FLC functions as part of a repressor complex together with another MADS-domain protein, SVP (Lee et al., 2007; Li et al., 2008).

During drought stress, one of the major morphological changes in angiosperms, is the early transition from vegetative to reproductive phase in order to complete their life cycle and make seeds before the stress conditions become too severe, leading to the plant's death. This mechanism of drought escape has been reported in several plant species (Xu et al., 2005; Franks et al., 2007; Franks, 2011; Su et al., 2013; Ma et al., 2014). In Arabidopsis, the onset of a drought escape response (in Col-0 and Ler-1 accessions) is controlled by the photoperiod; it is triggered by long day conditions (16 h light/ 8 h dark, LD) and slight but significantly repressed (only in Col-0) under short days (8 h light/16 h dark, also, short photoperiod, SD; Riboni et al., 2013). These opposite responses partially depend both on ABA, as ABA biosynthesis mutants flower later than wild type in control and in stress conditions, and on the expression of three MADS-box genes, *SOC1, FLC*, and *SVP* (Figure 1). Moreover, *SOC1* expression is induced by drought under a LD photoperiod in an ABA-dependent way; in addition, the *soc1-2* loss of function mutant strongly reduced the drought escape response under LD, and have a late flowering phenotype under this condition. On the other hand, under SD, the drought response is strongly dependent upon FLC / SVP complex repressor activity (Riboni et al., 2013, 2016). Under SD drought conditions, loss of function *svp-41* mutants recover the drought escape response (Figure 2D) and *flc-6* mutants show no alteration in their flowering time. Riboni et al. (2013) proposed that the adaptive significance for the interaction between drought and photoperiodic conditions relies upon limiting the floral transition to drought episodes that occur only in spring LD when environmental conditions allow plants to fulfill their life cycle and preventing them from occurring during SD of autumn, when environmental conditions are not favorable.

ROS are normally produced in mitochondria and chloroplasts by specific enzymes. Although these molecules may function in signaling, they could also be harmful for many biomolecules (Birben et al., 2012). One of the strategies that cells use to cope with oxidative stress is the synthesis of antioxidant enzymes such as peroxidases (Birben et al., 2012). PRX17 is a class III peroxidase present in all plant tissues, especially in young floral buds; intracellularly, it localizes in the cell wall and is important for lignin biosynthesis (Cosio et al., 2017). It has also been shown that PRX17 functions as a flower promoter, as *PRX17* loss of function impairs the transition to flowering while the OE line of this gene exhibits a slightly early flowering phenotype. Interestingly, the OE of the MADS-box gene *AGL15* is late flowering and the protein binds to PRX17 promoter, repressing its expression. Moreover, AGL15 not only regulates peroxidase expression but also the protein activity suggesting that part of the late flowering phenotype of the OE of *AGL15* depends on the repression of *PRX17* (Cosio et al., 2017). Additionally, SlMBP11, the AGL15 ortholog from tomato (see section 2.1), positively regulates the expression of catalase and other peroxidases, suggesting a general function of AGL15 and its orthologs as ROS regulators.

Cold stress responses occur after a short exposure to either chilling (<20°C) or freezing (<0°C) conditions (Chinnusamy et al., 2007). Under these stressful conditions, plants show different phenotypes such as chlorosis, reduced leaf expansion, poor germination, and delayed flowering (Barah et al., 2013; Jeon and Kim, 2013). Low temperature stress induces fast transcriptional responses in plants, characterized by the activation of the cold response pathway ICE-CBF-COR (ICE: inducer of CBF expression; CBF: C-Repeat Binding Factor, and COR: Cold Regulated) that, in turn, regulates more than 100 target genes needed to withstand cold (Seo et al., 2009; Wang et al., 2017).

The MADS-box gene *SOC1* not only integrates diverse floral inductive pathways (Boss et al., 2004) but also participates in a crosstalk between cold sensing and flowering. SOC1 negatively regulates cold response genes; for instance, the loss of function of this gene (*soc1-2*) induces the expression of several COR and CBFs genes, while in the OE line (soc1-101D), these genes are repressed (Seo et al., 2009). Chromatin Immunoprecipitation analysis showed that SOC1 repressed directly the expression of the three CBF genes (Seo et al., 2009). As mentioned, CBFs are known TFs that coordinate the expression of many genes related to cold stress responses, their OE in Arabidopsis not only activates COR genes but also FLC expression (a negative regulator of SOC1) and causes late flowering as well as tolerance to freezing conditions (Gilmour et al., 2004; Seo et al., 2009). Interestingly, these cross regulation, creates a loop between cold response signaling and flowering regulation (Figure 1).

Other MADS-domain proteins that negatively regulate the expression of *FT* and the transition to flowering in response to changes in temperature in Arabidopsis are SVP and FLOWERING LOCUS M (FLM, also known as MAF1 and AGL27). FLM, as SVP, represses the expression of the FPIs and functions as a negative regulator of flowering at low-temperature conditions (16°C, Lee et al., 2007, 2013; Posé et al., 2013). Interestingly, the *FLM* transcript shows alternative splicing with a different number of splice variants that depend on temperature growth conditions and the plant ecotype. Arabidopsis Col-0 has two splice variants that are, translated; the FLM-β delays flowering when overexpressed, whereas the OE of FLM-δ induces early flowering (Figure 1; Scortecci et al., 2003; Posé et al., 2013). Posé et al. (2013) showed that the relative accumulation of the two splice variant transcripts in Col-0 is also temperature-dependent, with FLM-β being the predominant form at 16°C while FLM-δ is the most abundant variant at 27°C. The data lead to the hypothesis that these two variants compete for the interaction with SVP at both temperatures. Moreover, it has been shown that SVP/FLM-β heterodimer is able to bind DNA, whereas the SVP/FLM-δ complex is impaired in DNA binding and may function as a dominant negative isoform. However, recent studies contradict this hypothesis by demonstrating that FLM-δ does not exercise a dominant-negative effect (Capovilla et al., 2017; Melzer, 2017). Additionally, other studies showed that at 16°C the splice variant FLM-β is more abundant than FLM-δ, but at 27°C the copy number of both splice variants was the same (Lee et al., 2013); and it was also shown that the level of FLM-β rather than the FLM-β/FLM-δ ratio controls flowering responses to high temperature (Figure 1; Sureshkumar et al., 2016; Lutz et al., 2017). Furthermore (Lee et al., 2013), have shown that the regulation of flowering upon temperature sensing depends also on the degradation of the SVP protein at high temperatures (Lee et al., 2013).

## 3. MADS-Box Genes Are Key in Modulating Developmental Responses to Seasonal Temperature Changes

Plant responses to various environmental conditions can alter the timing of initiation and the duration of different developmental events; moreover, the transition to different plant developmental processes, such as flowering, seed dormancy and germination, and bud dormancy and release, require specific environmental changes to occur. For example, many plants require a process known as vernalization (a prolonged exposition to cold conditions) to optimize flowering time with environmental conditions that ensure the maximum fitness (Sung and Amasino, 2005; Bäurle and Dean, 2006; Shu et al., 2016; Whittaker and Dean, 2017). In this section, we will focus on the participation of several MADS-box genes in seasonal low temperature-dependent developmental processes such as vernalization, seed germination, and bud dormancy and release.

### 3.1. Vernalization

Arabidopsis vernalizes at a wide range of temperatures (0–16°C) (Wollenberg and Amasino, 2012; Duncan et al., 2015), and the process leads to cold-induced epigenetic silencing of *FLC* (Figure 1; Bloomer and Dean, 2017). FLC is a protein that represses flowering transition by repressing flowering gene promoters, such as *FT* and *SOC1*. Upon vernalization, *FLC* expression and protein levels decrease so the FPIs are expressed and flowering is induced (Michaels and Amasino, 1999, 2001; Sheldon et al., 1999, 2000; Johanson et al., 2000; Rouse et al., 2002; Sung and Amasino, 2005; Whittaker and Dean, 2017). Moreover, repression of *FLC* involves epigenetic changes in histones, specifically an enrichment of trimethylated H3 lysine 27 (H3K27me3) and depletion of trimethylated H3 lysine 4 (Finnegan and Dennis, 2007; Yang et al., 2014).

Furthermore, and according to the data shown above, CBF transcription factors induce the expression of *FLC*, and this could explain the late-flowering phenotype of plants growing in cold stress conditions. However, in vernalization treatments where plants are exposed to prolonged cold conditions, CBFs genes are upregulated but *FLC* expression is inhibited. This suggests that vernalization reverses the cold stress CBFs' induction over *FLC* expression and that these two treatments affect flowering transition via distinct mechanisms (Seo et al., 2009).

In Arabidopsis, *FLC* has five paralogs (*MADS AFFECTING FLOWERING, MAF1* to M*AF5*), whose proteins show between 53 to 87% of identity (De Bodt et al., 2003; Ratcliffe et al., 2003). Similar to *FLC*, almost all *MAF* genes are regulated by vernalization: *FLM* (*MAF1*), *MAF2*, and *MAF3* are repressed, whilst *MAF5* is induced and *MAF4* is not affected (Ratcliffe et al., 2001, 2003). The loss of function mutant of *FLM* (*flm-1*) has an early flowering phenotype, while the OE of this gene retards flowering (Figure 1; Ratcliffe et al., 2001; Scortecci et al., 2001). In addition, MAF2 is involved both in the vernalization process (Ratcliffe et al., 2003) and in the flowering transition of plants exposed to short treatments of cold stress. The loss of function mutant of this gene (*maf2*) flowered disproportionately early when growing in short-duration cold treatments that do not elicit full vernalization in wild type plants; besides, regulation by *MAF2* was shown to be independent of *FLC* expression. These data suggest that MAF2 regulates the repression of premature vernalization in response to brief cold treatments (Ratcliffe et al., 2003). In the Landsberg accession, the OE of *FLC* and of *MAF1-MAF5* produced late-flowering lines (Michaels and Amasino, 1999; Ratcliffe et al., 2003), and the OE lines of *MAF2* are unable to respond to vernalization due to a continuous repression of *SOC1* (Ratcliffe et al., 2003).

The molecular mechanism activated in Arabidopsis in response to vernalization is conserved in temperate cereals (Greenup et al., 2010; Ruelens et al., 2013). In this group, vernalization response is regulated by the MADS-box gene *VERNALIZATION1* (*VRN1*) in wheat and by its ortholog *Vrn1-H1* in barley (Trevaskis et al., 2003). These genes are flowering promoters induced by vernalization, in an opposite manner to *FLC* (Danyluk et al., 2003; Yan et al., 2003; Sasani et al., 2009). In wheat and barley, the expression of *VRN1* genes, in plants not exposed to vernalization, depends on the cultivar and its requirements: high in spring lines that flower without vernalization, moderate in semi-spring lines, and null in winter lines that require vernalization to flower. Accordingly, the expression of *VRN1* in plants under vernalization correlates with the lines used: strongly induced in winter lines and weakly induced in spring lines (Trevaskis et al., 2003).

In plant perennial species, flowering continues throughout the lifespan of the plant, alternating vegetative and reproductive development. Interestingly, in *Arabis alpina*, a perennial plant, the expression of the *FLC* ortholog *PERPETUAL FLOWERING 1* (*PEP1*) depends on the external temperature, being upregulated in warm temperatures and downregulated in cold environments (Figure 3). Moreover, and contrary to what happens with *FLC, PEP1* expression is high after a vernalization treatment, correlating with the absence of H3K27me3 marks (Wang et al., 2009). Functional analysis using the loss of function mutant of PEP1 (*pep1*) showed that this gene is important to prevent flowering before vernalization and to facilitate the return to vegetative development, thus, restricting the duration of flowering. In addition, the OE of *PEP1* lines were late-flowering. These data suggest that *PEP1* expression is one of the mechanisms that *A. alpina* uses to perceive external temperature to be able to transit between flowering and vegetative development (Wang et al., 2009).

*CO* and *FT*-like genes have also been identified in barley and some grass species but their functions in flowering have not been described (Turner et al., 2005; King et al., 2006; Yan et al., 2006; Faure et al., 2007). Finally, another MADS-box gene with weak similarity to *SOC1, HvOS2* was found in barley (*Hordeum vulgare*) and its transcript levels decrease during vernalization in a pattern similar to that of *FLC* in Arabidopsis (Figure 3). However, although this gene does not contain the H3K27me3 deposition mark, its OE (*HvOS2*) delays flowering (Greenup et al., 2010).

### 3.2. Low Temperature-Dependent Germination

*FLC* is not only important for flowering but also participates in seed germination in response to seasonal environmental factors such that higher levels of *FLC* in OE lines, or in Arabidopsis accessions with different levels of *FLC*, provoke significantly elevated rates of germination at cool temperatures (10°C) compared to those at 22°C (Chiang et al., 2009). Moreover, it was also demonstrated that this *FLC* phenotype is dependent on the levels of ABA and GA, the two most important hormones for the initial stages of germination. In accessions with a strong *FLC* allele or in the OE lines of this gene, the expression of *CYP707A2*, a gene that participates in the catabolism of ABA, and *GA20ox1*, a gene that participates in the synthesis of GA, are upregulated. Additionally, and according to FLC-dependent germination models, the mutants of two other MADS-box genes related to flowering transition, *APETALA1* (*ap1-1*) and *soc1*, showed higher germination percentages at cool temperatures than wild type plants (Chiang et al., 2009).

### 3.3. Bud Dormancy and Release

Dormancy could be seen as a survival strategy during periods where the environmental conditions are adverse for growth. In perennial plants of temperate climates, the induction of bud dormancy by winter cold temperature and SD is a phenological adaptive feature which ensures optimal protection of vegetative and reproductive meristems against unfavorable environmental conditions (Bielenberg et al., 2008; Ríos et al., 2014). This latent state implies a reduction in meristematic cell proliferation activity; in addition, during the initial steps of dormancy formation, there is an increase in ABA levels and accumulation of storage compounds, as well as an active gene regulation engaged in the acquisition of desiccation and cold tolerance (Rohde and Bhalerao, 2007; Ruttink et al., 2007). Furthermore, SD induces bud dormancy by activating the ABA response (Singh et al., 2019). On the other hand, dormancy release requires resuming cell division, changes in some developmental programs regarding hormone accumulation, sugar metabolism, and epigenetic regulation. Moreover, for this transition to occur, exposition to a certain period of chilling temperatures is required, which could act as an active biological regulator more than as a stress signal (Horvath et al., 2003; David Law and Suttle, 2004; Rohde and Bhalerao, 2007; Liu et al., 2015).

Interestingly, the process of bud dormancy release in perennials presents similarities with the vernalization mechanism for flowering in Arabidopsis and cereals (Considine and Foyer, 2014); both processes require the occurrence of an extended chilling period, they are affected by photoperiod, and, in both processes, MADS-domain TFs are key regulatory elements (Chouard, 1960; Horvath et al., 2003; Hemming and Trevaskis, 2011). The MADS-box genes *DORMANCY-ASSOCIATED MADS-box* (*DAM*) have been implicated in regulating bud dormancy induction and release in woody plants. The first study made on peach (*Prunus persica*) indicated the participation of a cluster of six tandemly repeated *PpDAM1*-*PpDAM6* genes that are orthologs to *SVP* and *AGAMOUS LIKE24* from Arabidopsis (Bielenberg et al., 2004, 2008; Jiménez et al., 2009). Deletion of all six DAM genes in the *evergrowing* peach mutant provokes a constant growth of terminal meristems facing winter conditions, and in SD, the plants are also unable to form buds, displaying half the frost hardiness shown by wild type dormant trees (Figure 3; Rodriguez-A et al., 1994; Bielenberg et al., 2008).

Furthermore, there is a lot of molecular evidence that shows that *DAM*-like genes are differentially regulated during dormancy induction and release in various plant species (Leseberg et al., 2006; Mazzitelli et al., 2007; Horvath et al., 2008, 2010; Yamane et al., 2008, 2011; Hedley et al., 2010; Ubi et al., 2010; Sasaki et al., 2011; Wu et al., 2012; Saito et al., 2013). Additionally, *DAM* genes have been identified in quantitative trait loci that affect bud dormancy (Ruttink et al., 2007; Fan et al., 2010; Rohde et al., 2011; Sánchez-Pérez et al., 2012; Romeu et al., 2014; Zhebentyayeva et al., 2014).

Finally, the OE of DAM genes yield different developmental alterations. For example, the OE of *DAM6* in transgenic plums (*Prunus domestica*) results in plants with some degree of dwarfing and increased branching (Fan et al., 2010), and the OE of the *PmDAM6* in a poplar hybrid (*Populus tremula* x *Populus tremuloides*) promotes growth cessation and dormancy onset under favorable conditions (Sasaki et al., 2011). Also, the OE of a DAM ortholog of apple (35S:*MdDAMb*; *Malus domestica*, “Royal Gala”) delays the timing of spring budbreak and produces plants displaying a dominant main stem with few lateral shoots (Wu et al., 2017a). Noteworthily, Horvath et al. (2008) suggested that the interaction between DAM proteins and FT is fundamental for dormancy transitions, resembling the interactions among these genes' orthologs during flowering transition.

There are still some other MADS-box genes whose expression changes during dormancy transitions and that have been functionally characterized (Horvath et al., 2008). For instance, the ortholog of *SVP* (a repressor of flowering) of aspen plants, *SHORT VEGETATIVE PHASE-LIKE* (*SVL*), is downregulated by exposure to low temperatures, upregulated by ABA, and induced by SD in an ABA-dependent pathway (Singh et al., 2018, 2019). Moreover, loss of function of *SVL* plants (*SVL*-RNAi) showed early budbreak, while OE plants display late budbreak compared to wild type plants (Singh et al., 2018). In addition, the OE of *SLV* in *abi1-1* loss of function mutants rescues dormancy regulation (Singh et al., 2019). SVL directly and positively regulates the expression of an enzyme that participates in callose deposition, *CALLOSE SYNTHASE 1*, involved in plasmodesmatal closure important to promote dormancy (Singh et al., 2019). Besides, it negatively regulates the expression of one of the *FT* poplar orthologs (*FT1*) that participates in dormancy release (Busov, 2019).

The *SVP* ortholog is also important for bud dormancy release in kiwifruit (*Actinidia* spp.). This plant requires a minimum number of chilling hours so that buds can reinduce growth; however, chilling requirements vary among different species (Lionakis and Schwabe, 1984; Snelgar et al., 2007). Ectopic expression of *SVP2* in a low-chill kiwifruit *Actinidia eriantha* had a minimal effect on the duration of dormancy while it greatly affected the duration of dormancy in a high-chill kiwifruit *A. deliciosa* (Figure 3; Wu et al., 2012, 2017b).

Also, in apple, the ectopic expression of an ortholog of *SVP* (35S:*MdSVPa*) delayed bud release (Wu et al., 2017a). Furthermore, there are some reports of *SVP* ortholog regulation in different plants during the onset and release of dormancy (Mazzitelli et al., 2007; Yamane et al., 2008; Diaz-Riquelme et al., 2009; Horvath et al., 2010; Wu et al., 2012).

In addition, it has also been shown that the OE of the *FUL*-like gene of *Populus* in birch (*Betula pendula*) resulted in delayed dormancy (Hoenicka et al., 2008). Moreover, a *SOC1*-like gene was upregulated in poplar (*Populus tremula* x *Populus alba*) during bud formation when induced by variations in the photoperiod (Ruttink et al., 2007). Additionally, a *SOC1*-like gene was expressed at higher levels relative to other tissues in developing buds of white spruce (*Picea glauca*), implying that this MADS-box could play a specialized role in bud development (El Kayal et al., 2011). Also, different allelic variants of the *SOC1*-like gene of apricot are associated with different chilling requirements for dormancy release (Trainin et al., 2013).

Nevertheless, despite all these works, little is known about how MADS-box genes contribute to the maintenance of bud dormancy and dormancy release, functional investigations are still required to determine the role played by DAM and other MADS-box genes in the activity-dormancy cycle, together with the identification of their target genes (Cooke et al., 2012). Even so, these data support the parallelism between regulation of bud dormancy and vernalization.

## 4. Conclusions

Alterations in gene regulation are among the most significant mechanisms for phenotypic change (Hoekstra and Coyne, 2007); within these, TFs can function as developmental switches given their capacity to reprogram gene expression. It has also been shown that TFs are important in regulating plant responses to environmental stress along their lifespan. Moreover, it has been shown that small changes in some key TFs determine the evolution of different processes and structures. Specifically, TFs of the MADS-box gene family are central regulators of every aspect of Arabidopsis development as shown by mutant analysis. Several data also suggest that distinct MADS-box genes not only alter their expression levels when facing different stress conditions but that they are involved in mediating plant responses or tolerance to a wide range of abiotic stresses, addressing their importance as integrators of environmental cues and endogenous hormones in a taxonomically broad range of plant species. Some MADS-box genes act as critical negative regulators of growth improving plant survival, while others function as positive regulators of stress tolerance, associated with regulating the maintenance of primary metabolism, ABA signaling, ROS homeostasis, and detoxification processes through antioxidant enzymatic activities. Despite these studies, many mechanisms whereby MADS-box genes coordinate the transcriptional response to abiotic stress remain to be identified.

## Author Contributions

NC-M, JH, and AG-A conceived and wrote the paper. WC-S, MA, CT, BG-P, MS, and EA-B wrote the paper. All authors read and approved the final version.

### Conflict of Interest Statement

The authors declare that the research was conducted in the absence of any commercial or financial relationships that could be construed as a potential conflict of interest.
